# An Osteosarcoma Model by 3D Printed Polyurethane Scaffold and In Vitro Generated Bone Extracellular Matrix

**DOI:** 10.3390/cancers14082003

**Published:** 2022-04-15

**Authors:** Nicola Contessi Negrini, Claudio Ricci, Federica Bongiorni, Luisa Trombi, Delfo D’Alessandro, Serena Danti, Silvia Farè

**Affiliations:** 1 Department of Chemistry, Materials and Chemical Engineering “G. Natta”, Politecnico di Milano, 20131 Milan, Italy; federica.bongiorni@mail.polimi.it (F.B.); silvia.fare@polimi.it (S.F.); 2 Department of Civil and Industrial Engineering, University of Pisa, 56122 Pisa, Italy; claudio.ricci@med.unipi.it; 3 Department of Surgical, Medical, Molecular Pathology, University of Pisa, 56126 Pisa, Italy; l.trombi@yahoo.it (L.T.); delfo.dalessandro@unipi.it (D.D.)

**Keywords:** fused deposition modeling, cancer tissue engineering, in vitro model, mechanical properties, mesenchymal stromal cell, bone matrix, bone cancer, tumor microenvironment

## Abstract

**Simple Summary:**

Development of new therapeutics to treat osteosarcoma is fundamental to decreasing its current health impact. 3D in vitro models are gaining tremendous momentum as, compared to traditional 2D in vitro models and in vivo models, can speed up new treatment discovery and provide clarification of the pathology development, by ultimately offering a reproducible and biomimetic tool. However, engineering a 3D osteosarcoma in vitro model is challenging, since the reliability of the models strictly depends on their ability to correctly mimic the physical, mechanical, and biological properties of the pathological tissue to be replicated. Here, we designed 3D printed polyurethane scaffolds enriched by in vitro pre-generated bone extracellular matrix, synthesized by osteo-differentiated human mesenchymal stromal cells, to replicate in vitro an osteosarcoma model, which can be potentially used to study tumor progression and to assess new treatments.

**Abstract:**

Osteosarcoma is a primary bone tumor characterized by a dismal prognosis, especially in the case of recurrent disease or metastases. Therefore, tools to understand in-depth osteosarcoma progression and ultimately develop new therapeutics are urgently required. 3D in vitro models can provide an optimal option, as they are highly reproducible, yet sufficiently complex, thus reliable alternatives to 2D in vitro and in vivo models. Here, we describe 3D in vitro osteosarcoma models prepared by printing polyurethane (PU) by fused deposition modeling, further enriched with human mesenchymal stromal cell (hMSC)-secreted biomolecules. We printed scaffolds with different morphologies by changing their design (i.e., the distance between printed filaments and printed patterns) to obtain different pore geometry, size, and distribution. The printed PU scaffolds were stable during in vitro cultures, showed adequate porosity (55–67%) and tunable mechanical properties (Young’s modulus ranging in 0.5–4.0 MPa), and resulted in cytocompatible. We developed the in vitro model by seeding SAOS-2 cells on the optimal PU scaffold (i.e., 0.7 mm inter-filament distance, 60° pattern), by testing different pre-conditioning factors: none, undifferentiated hMSC-secreted, and osteo-differentiated hMSC-secreted extracellular matrix (ECM), which were obtained by cell lysis before SAOS-2 seeding. Scaffolds pre-cultured with osteo-differentiated hMSCs, subsequently lysed, and seeded with SAOS-2 cells showed optimal colonization, thus disclosing a suitable biomimetic microenvironment for osteosarcoma cells, which can be useful both in tumor biology study and, possibly, treatment.

## 1. Introduction

The development of three-dimensional (3D) tissue or organ biomimetic models has become a topic of major interest in the field of biomaterials and tissue engineering. In vitro bioartificial 3D models are unique tools to study the development of pathologies and test potential new drugs [1], to better understand tissue and organ morphogenesis [2], and to recapitulate tumorigenesis and cancer progression to potentially develop new personalized treatments and therapeutics for precision medicine [3]. In the latter case, cancer tissue engineering has emerged in the bioengineering field aiming at fabricating 3D in vitro models to mimic the multi-dimensional structure, organization, and complex relationship in diverse tumor microenvironments (TMEs) [4]. Conventional studies to assess therapeutic efficacy on tumor cells have usually been performed using traditional two-dimensional (2D) in vitro models and small animal in vivo models. However, a wide consensus has now been reached on the evidence that 2D models lack mimicking structural, mechanical, and biochemical cues of the tissue microenvironment, thus oversimplifying cell-cell and cell-extracellular matrix (ECM) interactions [5]. Moreover, animal models are affected by several limitations, including low reproducibility, the impossibility of performing longitudinal studies due to animal sacrifice at the end of most tests, associated high costs, time-consuming studies, as well as ethical concerns and the necessity of ethical approvement by dedicated bodies [3]. 3D in vitro models represent an optimal compromise between the use of 2D in vitro models and in vivo models, as they mimic the complex 3D architecture of tissues and they can recapitulate the cell-cell and cell-ECM interactions, thus leading to improved predictions [6,7]. Despite undeniable advantages, the fabrication of 3D in vitro models is still challenging for bioengineers, as prepared models should possess adequate properties to mimic the specific tissue and pathology to be bioartificially recreated [8].

Osteosarcoma is an osteoid-producing primary malignant bone tumor of mesenchymal origin, primarily affecting long bones of the extremities, with the highest incidence in the distal femur, proximal humerus, and proximal tibia [9]. Despite being relatively rare, race and age have been identified as risk factors, with those aged 10–14 years old and over 65 years old being the most affected [10]. Current treatments for osteosarcoma include surgical resection of the diseased portion, coupled with systemic chemotherapy. However, differently from carcinomas (epithelial origin), the safe resection margin in osteosarcoma resection is still unknown due to the unconfined structure of this tumor. As such, the 5-year event-free survival for patients with early diagnosed localized osteosarcoma is 65–70%, whereas patients with recurrent disease or metastases, most commonly in the lungs (85–90%), have a very poor prognosis of approximatively 20–30% survival rate [11,12]. The development of tools to allow a deep understanding of the tumor evolution and its interaction with the tissue microenvironment is thus impellent to identify molecular targets, anticancer drugs, and patient-specific treatments, which would improve the current pathological outcomes.

Typical steps used to study the efficiency of drugs and treatments avail themselves of 2D in vitro cell cultures, followed by in vivo animal models, and finally pre-clinical and clinical trials. However, most of the drugs/treatments demonstrated to be efficient on 2D in vitro models fail in replicating this high efficiency in vivo, given the oversimplification of the 2D models, thus leading to a waste of resources [13]. 3D in vitro osteosarcoma models are required to speed up new therapeutic evaluation and they are the objective of several studies. Such advanced osteosarcoma models can be distinguished in: *(i)* scaffold-free systems (e.g., multicellular tumor spheroids and organoids cultured in suspension), and *(ii)* scaffold-based cancer tissue engineering approaches. In the latter case, the fabrication of biomaterial scaffolds to support the 3D tumor development allows reproducible and easy to handle culture systems to be obtained, provided with tunable biochemical/mechanical properties, which are in turn capable of mimicking those of the native tumor microenvironment [14,15]. To date, the scientific literature mostly reports on 3D scaffolds to study secondary tumors in bone. Several studies describe scaffolds designed to mimic in vitro the development of breast cancer bone metastasis, including polyurethane (PU) foams [16], porous chitosan scaffolds loaded with hydroxyapatite [17], silk fibroin scaffolds [18,19], poly(lactide-co-glycolide) (PLGA) porous scaffolds mineralized with hydroxyapatite [20], 3D hyaluronate-based hydrogels [21], and polyethylene glycol (PEG) hydrogels coupled with printed polycaprolactone (PCL) [22]. Only a few works describe the development of scaffolds addressing in vitro models of primary bone tumors. Electrospun poly(ε-caprolactone) scaffolds were used to prepare Ewing osteosarcoma in vitro models [23,24], porous silk sponges were fabricated via freeze-drying, seeded with osteosarcoma cells, and the prepared model was compared to traditional 2D in vitro cultures and in vivo mice models [25], and methacryloyl platelet lysate was used to sustain spheroid growth and invasion [26]. Nevertheless, replicating in vitro a biomimetic microenvironment of bone cancer in terms of morphological, mechanical, chemical, and biological cues represents a challenge [27]. Among the plethora of technologies used for scaffold fabrication, 3D printing by fused deposition modeling is a subset of additive manufacturing techniques, with high control of geometric parameters, such as pore and filament size, fundamental to tailoring biomaterials properties. PU is a class of polymers entitled with diverse properties. PUs are widely known in the biomedical field for their applications in medical devices and have recently become attractive as 3D printing biomaterials [28]. Moreover, previous evidence highlighted the importance of biochemical signaling, often provided by artificial hydroxyapatite coatings of the polymeric surfaces [13,16]. A possible strategy, previously used in bone tissue engineering, relies on the application of bone cells in a first culture step to produce bone ECM and then to remove these cells by lysis to have a biohybrid scaffold in which to seed the stem cells of interest [29,30]. The advantage of this strategy is that a biomimetic microenvironment is generated, not only containing hydroxyapatite, but also bone proteins and growth factors secreted by non-pathogenic cells [31].

Here, we developed a biomimetic osteosarcoma in vitro model via 3D printed poly-ether-urethane (hereafter referred to as PU) scaffolds, post-decorated with human stromal cell (hMSC)-synthesized ECM. In the first phase of this study, we tested different printed geometries to achieve optimal morphological, physical, and mechanical properties to replicate a bone-like model and allow hMSC colonization and differentiation towards osteoblastic lineage. After selecting the most promising scaffolds, we developed different biomimetic models by the deposition of biomolecules synthesized by undifferentiated or osteo-differentiated hMSC. The constructs were then lysed to remove live cells and seeded with SAOS-2 osteosarcoma cells to investigate the potential use of the prepared scaffold as in vitro osteosarcoma model.

## 2. Materials and Methods

### 2.1. Materials

Thermoplastic PU (hardness: 80 Shore A) 3D printing filaments were purchased from Sharebot S.r.l. (Nibionno, Italy). Lymphoprep was purchased by GE healthcare (Hatfield, UK). Low-glucose Dulbecco’s Modified Eagles Medium (DMEM), L-glutamine, penicillin, streptomycin heat-inactivated fetal bovine serum, trypsin, CaCl_2_, human AB plasma, *p*-nitrophenol standards, alkaline buffer solution (1.5 M 2-amino-2-methyl-1-propanol at pH 10.3), 4-nitrophenyl phosphate disodium salt hexahydrate capsules, NaOH, acetic acid, phosphate-buffered saline (PBS), methanol, 36 vol-H_2_O_2_, bovine serum albumin (BSA), Mayer’s hematoxylin, dibutyl-phthalate polystyrene xylene (DPX), silver nitrate, pyrogallol, and sodium thiosulfate were obtained from Sigma-Aldrich, (Milan, Italy). The osteogenic differentiation medium was bought from Lonza (Basel, Switzerland). AlamarBlue™, PicoGreen kit, and Paraffin Histoplast LP were supplied by Thermo Fisher Scientific (Waltham, MA, USA). Arsenazo III was bought from Diagnostic Chemicals Ltd. (Oxford, CT, USA). Neutral buffered formalin, absolute ethanol, and xylene were purchased from Bio-Optica (Milan, Italy). Aluminum sulfate was obtained from Carlo Erba (Milan, Italy). Goat serum was obtained from Vector Lab (Burlingame, CA, USA). Primary antibody for collagen type I (COLL-1; ab34710) was bought from AbCam (Cambridge, UK). Primary antibodies for osteopontin (OPN; sc-166261), fibronectin (FN; sc-59826), transforming growth factor beta 1 (TGF-β1; sc-146), and alkaline phosphatase (ALP; sc-166261) were supplied by Santa Cruz Biotechnology, (Santa Cruz, CA, USA). Goat anti-rabbit and goat anti-mouse biotinylated secondary antibodies and streptavidin (Vectastain Elite ABC Kit Standard) were obtained from Vector Laboratories (Burlingame, CA, USA). SAOS-2 cell line (89050205) was bought from ATCC-LG standards (Milan, Italy). L929 (85011425) cells were obtained by ECACC (Salisbury, UK). McCoy’s 5A medium was bought from Gibco (Rodano, Italy). HMSCs were supplied by Merck Millipore S.A.S. (Burlington, MA, USA).

### 2.2. 3D Printing of PU Scaffolds

Computer-aided design (CAD) models were prepared by SolidWorks® software (Dossault Systems Solidworks Corp., Waltham, MA, USA). The CAD models were then processed by Slic3r software (open source) to obtain stl files. These files were used to print PU scaffolds by processing PU 3D printing filaments via fused deposition modeling (Sharebot 42, Sharebot s.r.l., Nibionno, Italy). Each scaffold was printed by subsequent deposition of 12 parallel layers (thickness = 4.8 mm) and scaffold specimens were subsequently cut with a scalpel (square shape, side = 6 mm). The angle shift between subsequent layers and the distance between the axis of the printed filaments were varied (i.e., 90°, 60°, and 45° angle shift between subsequent layers of parallel filaments printed at 1.0 mm or 0.7 mm distance) to obtain different scaffold types, as summarized in Table 1. The surface roughness of printed filaments (*n* = 6) was measured according to DIN 4768 (R_a_, R_z_Iso, UBM MICROFOCUS).

### 2.3. Morphological, Physical, and Mechanical Characterization

The morphological characterization of the printed PU scaffolds was performed by stereomicroscope (Leica DFC290, Leica Microsystems). Top-view images of the scaffolds (*n* = 6 images per *n* = 3 scaffolds per type) were acquired and analyzed by ImageJ software (version 1.52e). The average pore area for each scaffold type was calculated by measuring the area of pores in binary images. The density of the scaffolds was calculated as a mass-to-volume ratio of the samples (*n* = 5). The porosity percentage of each scaffold formulation was calculated according to EN ISO 4590, following Equation (1):

(1) $$\mathrm{Porosity}\;\left[\%\right]=(1\;-\;\frac{\rho_{\mathrm{scaffold}}}{\rho_{\mathrm{material}}})\;\times \;100$$

where ρ_scaffold_ is the density of the scaffolds, while ρ_material_ is the density of the PU samples printed with 100% infill.

The weight variation of the printed scaffold in a culture medium, tested to simulate their potential use as in vitro model, was evaluated after scaffold sterilization (immersion in absolute ethanol, exposure under UV light on both sides for 20 min). Dry samples (*n* = 3) were weighted (w_0_), immersed in a culture medium, and stored in an incubator to simulate the cell culture condition (37 °C, 5% CO_2_). At established time points, samples were removed from the culture medium and weighted (w_t_). The culture medium was refreshed every three days. The percentage weight variation in time (ΔW%) was then calculated as Equation (2) [32]:

(2) $$\Delta W\;\left[\%\right]=\frac{w_{t}{\;-\;w}_{0}}{w_{0}}\;\times \;100$$

Static compression tests were performed by Dynamic Mechanical Analyzer (DMA Q800, TA Instruments) to investigate the mechanical properties of the scaffolds. Hydrated samples (i.e., immersed until weight variation reached the plateau) were tested at 37 °C by applying a load ramp at 0.5 N∙min^−1^ (0.05 N preload) up to 18 N (i.e., load limit of the machine). The Young modulus E was calculated as the slope of the curve in the 0–1% strain range (R^2^ > 0.9).

### 2.4. In Vitro Cytotoxicity Tests on PU Scaffolds

Samples printed with a 0.7 mm distance between filaments were selected for the in vitro tests. Samples were sterilized by immersion in absolute ethanol and by exposure to UV light (20 min per side).

In vitro, indirect cytotoxicity tests (according to UNI EN ISO 10993) were performed by using L929 and SAOS-2 cells. Scaffold samples (*n* = 3) were lodged in 24-multiwell tissue culture polystyrene (TCPS), immersed in a 1.2 mL culture medium, and stored in an incubator (37 °C, 5% CO_2_) for 1, 3, and 7 days. At each time point, a culture medium without samples was placed in the incubator as a control. L929 and SAOS-2 cells were seeded in 96-multiwell TCPS at 1 × 10^4^ cells/well density and cultured until 70% confluency was reached. Then, the culture medium was replaced with the scaffold supernatant to continue cell cultures for 24 h, using a non-conditioned culture medium as control. The cell metabolic activity was evaluated using the AlamarBlue™ assay, as Equation (3) [33]:

(3) $$\mathrm{Cell}\;\mathrm{Viability}\;\left[\%\right]=\frac{f_{\mathrm{eluate}}{\;-\;f}_{\mathrm{AlamarBlue}}}{f_{\mathrm{control}}{\;-\;f}_{\mathrm{AlamarBlue}}}\;\times \;100$$

where f_eluate_ is the fluorescence intensity of the supernatant of the cells cultured with culture medium eluates, f_control_ the intensity of the supernatant measured for cells incubated with culture medium controls, and f_AlamarBlue_ the background fluorescence of the unreacted AlamarBlue solution (λ_excitation_ = 540 nm, λ_emission_ = 595 nm, using a UV spectrophotometer (Tecan GENius Plus plate reader, Tecan Trading AG, Männedorf, Switzerland).

### 2.5. 3D Bone Model Preparation

The suitability of the printed scaffolds as 3D in vitro culture systems for bone was first evaluated by seeding and then osteo-differentiating hMSCs on scaffolds printed with 90°, 60°, and 45° angle shift patterns. HMSC was expanded in a culture medium consisting of low-glucose DMEM added with 2 mM L-glutamine, 100 IU/mL penicillin, 100 mg/mL streptomycin, and 10% heat-inactivated FBS until 70–80% confluence was reached. HMSCs at 500.000 cells/sample were resuspended in 50 μL of human AB plasma with 20 μL CaCl_2_ 25 mM, seeded on 90_0.7, 60_0.7, and 45_0.7 printed PU scaffolds, and incubated for 30 min at 37 °C in 24-multiwell TCPS. Subsequently, 1 mL of culture medium was added to each sample. The following day, the expansion medium was changed to an osteogenic differentiation medium, and the culture was continued for 2 weeks by replacing the culture medium every 2–3 days. The metabolic activity of hMSC/PU scaffold constructs was measured by AlamarBlue assay. On days 1, 7, and 14 of culture, the samples (*n* = 3) were incubated for 3 h at 37 °C with the dye diluted in the culture medium as per the manufacturer’s instruction. For each test, 100 μL of supernatant from samples or controls were loaded in 96-multiwell TCPS and the absorbance was measured by spectrophotometry (Victor 3; PerkinElmer, Waltham, MA, USA) under double wavelength reading (i.e., at 570 nm and 600 nm). The AlamarBlue percentage (%AB_red_) was calculated by the Equation (4), as for the manufacturer’s protocol, in which: λ = absorbance, s = sample, and c = control:

(4) $${\%\mathrm{AB}}_{\mathrm{red}}=\frac{\left(117,216\cdot \lambda_{s\left(570\;\mathrm{nm}\right)}-80,586\cdot \lambda_{s\left(600\;\mathrm{nm}\right)}\right)}{\left(155,677\cdot \lambda_{c\left(600\;\mathrm{nm}\right)}-14,652\cdot \lambda_{c\left(570\;\mathrm{nm}\right)}\right)}\;\times \;100$$

Cellularity (i.e., DNA content) in construct lysates was quantified by PicoGreen assay. Constructs (*n* = 3) previously frozen in 2 mL of double-distilled (dd)-water underwent three freeze/thaw cycles followed by vortexing to allow DNA extraction from the samples. DNA standards (range: 0–6 μg/mL) were prepared and 50 μg/mL of standard or sample was poured into individual wells of a 96-multiwell plate. Working buffer and PicoGreen dye solution, prepared following the manufacturer’s instructions, were added at 100 μg/mL and 150 μg/mL per well, respectively. After 10 min incubation in the dark at room temperature (RT), the fluorescence intensity was measured at λ_excitation_ = 480 nm, λ_emission_ = 520 nm, using Victor 3 plate reader.

On the residual samples not used for cellularity (*n* = 3), intracellular ALP was quantified via a colorimetric end-point assay using *p*-nitrophenol phosphate as a reagent. The rate of *p*-nitrophenol formation, catalyzed by ALP, is directly related to the enzyme activity in the sample. *p*-nitrophenol standards (range: 0–250 μM) were prepared, and 80 μL of standard or sample was inserted into individual wells of a 96-multiwell plate. The alkaline buffer solution was added at 20 μL/well. Substrate solution was prepared by dissolving the capsules into dd-water with a final concentration of 4 mg/mL and added at 100 μL/well. The microplate was incubated at 37 °C for 1 h and the reaction was stopped by adding 0.3 M NaOH at 100 μL/well. The absorbance was measured at 405 nm using Victor 3 plate reader.

Finally, the amount of mineralized ECM present, in terms of calcium content, was quantified using the Arsenazo III reagent. 1 N acetic acid was added to equal volumes of residual lysate solutions to obtain 0.5 M acetic acid sample solutions. Samples (*n* = 3) were incubated overnight to dissolve calcium deposits. Ca^2+^ standards (range: 0–100 μg/mL) were prepared from dilutions of a 1 mg/mL stock solution of CaCl_2_ and 20 μL of standard or sample was added to individual wells of a 96-multiwell plate. MilliQ water solution at 300 μL/well of Arsenazo III > 0.15 M was added and the microplate was incubated for 10 min at RT. The absorbance at 650 nm was measured on a Victor 3 plate reader. Cell distribution and morphology on the scaffolds were investigated via histological analysis. The constructs were fixed in neutral buffered formalin (4% *w*/*v*) at 4 °C overnight, and washed in PBS. Dehydration was done using a graded series of ethanol aqueous solutions at 40 °C. The samples were clarified in xylene overnight at RT and rinsed in liquid paraffin in a vacuum oven at 60 °C for 24 h, placed in a wax embedding box, and air-dried. Paraffin blocks were sectioned by a microtome into 6 µm-thick sections that were mounted on glass slides. The sections were deparaffinized in xylene, rehydrated in absolute ethanol, washed in distilled water, and PBS×1. The specimens were incubated in a methanolic solution containing 0.6% *v/v* 36-volume H₂O₂, in the dark for 15 min to quench endogenous peroxidases. After washings in PBS, specimens were incubated with 5% *v/v* goat serum for 20 min at 37 °C to block a-specific binding sites of the secondary antibodies. Sections were then incubated with primary antibodies diluted in 0.1% *w/v* bovine BSA in a moist chamber overnight at 4 °C. The following antibodies were used: rabbit polyclonal anti-human collagen type I 1:1200 mouse monoclonal anti-human Osteopontin 1:2000 and mouse monoclonal anti-human Alkaline Phosphatase 1:100. Negative controls were obtained by incubating sections with only BSA/PBS solution. Specimens were then incubated with goat anti-rabbit or goat anti-mouse biotinylated secondary antibodies diluted 1:200 in 1.5% *v/v* goat serum-1x PBS solution for 60 min, then with streptavidin solution for 30 min, prepared according to the manufacturer’s instructions. Sections were incubated in the substrate-chromogen solution (0.5 mg/mL of 3,3′-diaminobenzidine tetrahydrochloride) and added with H₂O₂, for 5 min in the dark, then counterstained with Mayer’s hematoxylin for 2 min and washed in tap water for 2 min. Sections were dehydrated in absolute ethanol, clarified, and mounted with a coverslip DPX mounting medium. Stained sections were observed with a Nikon Eclipse Ci microscope (Nikon Instruments, Amsterdam, The Netherlands) equipped with a digital camera.

### 2.6. 3D Osteosarcoma Model Preparation and Validation

In vitro osteosarcoma models were prepared by seeding SAOS-2 cells on PU scaffolds printed with a 60° angle shift pattern. SAOS-2 cells (1 × 10^6^ cells) were resuspended in 50 μL of human AB plasma with 20 μL CaCl_2_ 25 mM and seeded on differently pre-treated PU scaffolds for 30 min at 37 °C in 24-multiwell TCPS. The different pre-treatments included *(i)* printed PU scaffolds (i.e., no pre-treatment), (*ii*) PU scaffolds containing non-mineralized ECM produced by pre-seeded undifferentiated hMSCs (PU_ECM), which were further lysed in sterile H_2_O, and *(iii)* PU scaffolds containing mineralized ECM produced by osteodifferentiated hMSCs, which were further lysed in sterile H_2_O (PU_bECM). The PU_ECM constructs were obtained as described in Section 2.5, either using an hMSC growth medium or an osteo-differentiating culture medium for 3 weeks. After cell seeding, 1 mL of McCoy’s 5A culture medium was added, and the constructs were cultured for 1 week. The different prepared models are summarized in Table 2.

The in vitro models were then processed for histological analysis, as previously described. H&E staining was performed to study cell morphology and scaffold colonization, as reported in Section 2.6. In addition, von Kossa staining was performed to reveal deposits of calcium phosphate, typical of bone mineralized ECM. After deparaffination and rehydration, sections were washed in PBS, incubated with 1% *w/v* silver nitrate exposed to light, 0.5% *w/v* pyrogallol, and 5% *w/v* sodium thiosulfate. The counterstaining was performed by incubating cells with 0,1% *w/v* nuclear fast red diluted in a distilled water solution containing 5% *w/v* aluminum sulfate, followed by washing in tap water to visualize the staining. Sections were dehydrated, clarified, mounted, and observed as previously described.

### 2.7. Statistical Analysis

Data were analyzed by GraphPad Prism software. All data are represented as mean ± standard deviation. Statistical analysis was performed by one-way ANOVA test and Tukey’s multiple comparisons, considering *p* < 0.05 as statistically significant.

## 3. Results

In vitro osteosarcoma, 3D models were developed by first optimizing the physicomechanical properties of the printed PU scaffolds for a bone 3D model. Different layouts of the PU scaffolds were obtained by changing the angle shift printing pattern and the distance between the filaments. The cytocompatibility of the produced scaffolds was assessed using SAOS-2 and L929. Thereafter, the most suitable scaffold allowing a 3D in vitro model of bone tissue was selected using osteo-differentiated hMSCs. The selected PU scaffold was then used to generate osteosarcoma 3D models by seeding SAOS-2 cells. Different treatments were preliminarily applied to the polymeric scaffold to recapitulate in vitro the development of osteosarcoma, aiming at understanding the role of biological molecules.

### 3.1. Morphological, Physical, and Mechanical Properties of 3D Printed Polyurethane Scaffolds

Optimization of the fabrication parameters and procedure was required to 3D print defect-less scaffolds with reproducible geometry. The initial printing trials achieved scaffolds characterized by morphological irregularities, given by insufficient extrusion of material and non-uniform deposition of the printed filaments. Thus, the printing parameters were adjusted to achieve a uniformly printed structure. The optimized printing parameters are summarized in Table 3. Afterward, the Gcode files used to print the scaffolds were modified by Repetier-Host software to guide the movements of the printing head, thus avoiding defects in the deposited material during the printing process. Optimization of the printing and design parameters and modification of the printing movements allowed scaffolds with desired and reproducible geometries to be finally obtained (Figure S1). These parameters were then applied to print scaffolds with different geometries and subsequently tested as potential in vitro osteosarcoma models.

**Figure 1:**
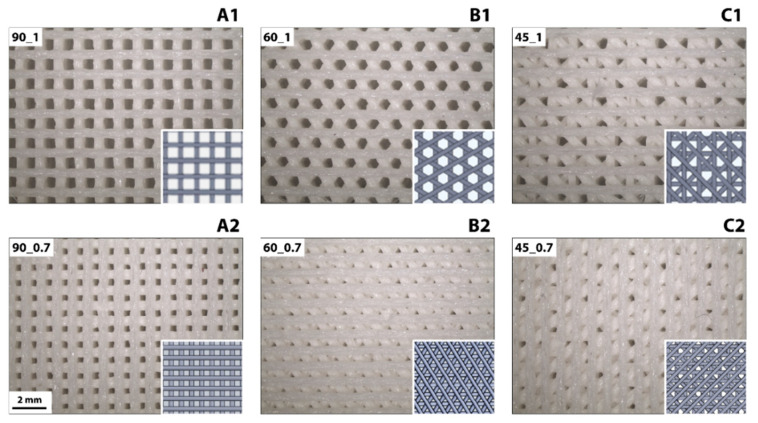
Morphology of 3D printed PU scaffolds imaged via stereomicroscope. Columns ((**A1**,**A2**,**B1**,**B2**,**C1**,**C2**)) show different printing patterns: 90°, 60°, and 45° angle shifts between subsequent printed layers, respectively. The top row (**A1**,**B1**,**C1**) shows a 1.0 mm distance between filaments, whereas the bottom row (**A2**,**B2**,**C2**) 0.7 mm distance between filaments. Scale bar = 2 mm for all. Inset images depict computer-assisted design (CAD) files used to print the scaffolds.

The printed PU filaments were characterized by roughness values R_a_ = 0.60 ± 0.20 μm and R_z_Iso = 4.09 ± 1.22 μm. Six different scaffold geometries (i.e., 90_1, 60_1, 45_1, 90_0.7, 60_0.7, and 45_0.7) were printed by varying the printing pattern (i.e., 90°, 60°, and 45° angle shift between subsequent printed layers) and distance between filaments (i.e., 1.0 mm and 0.7 mm). Representative images of the printed patterns compared to the computer-assisted design (CAD) drawing set for the printing process are shown in Figure 1**.**

All the printed geometries reproduced the CAD designs used to print the scaffolds. The measured distance between the center of printed filaments corresponded to the one set in the different CAD models (i.e., for all the printed geometries, error < 1%), proving the correct deposition of filaments following the CAD model. The average diameter of the printed filaments was 0.57 ± 0.07 mm, approximatively 40% higher than the set filament thickness. This increase in thickness was attributed to the printing process that melted the filament, thus causing it’s partial spreading upon deposition. The latter is a well-studied phenomenon occurring during fused deposition modeling printing procedures [34]. Different pore architectures were achieved by varying the angle shift between printed layers (Figure 1(A1,A2,B1,B2,C1,C2)) and the distance between adjacent filaments (Figure 1(A1,A2,B1,B2,C1,C2)). SEM micrographs displaying the printed geometries and surface of the printed filaments are displayed in Figure S2. Scaffolds printed with 90° shift angle patterns showed squared pores, for both 1.0 mm and 0.7 mm inter-filament distances. Scaffolds printed with 60° shift angle patterns had hexagonal pores in case of 1 mm inter-filament distance, whereas triangular pores in case of 0.7 mm inter-filament distance. Scaffolds printed with 45° shift angle patterns showed pores with a more complex, rose-like shape. For all the considered geometries, no structure collapse was observed, as confirmed by the porous structure observed for the cross-sections of the 3D printed PU scaffolds (Figure S3). 

**Figure 2:**
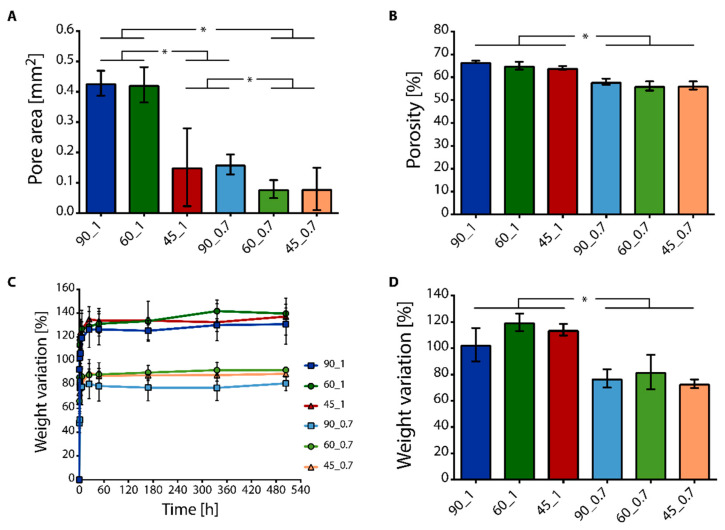
Physical characterization of 3D polyurethane (PU) scaffolds. (**A**) Average pore area determined by top-view images of the printed scaffolds (* *p* < 0.05), and (**B**) percent porosity (* *p* < 0.05). (**C**) Weight variation of the printed PU scaffolds after immersion in culture medium, and (**D**) weight variation at plateau (* *p* < 0.05).

The physicomechanical properties of the scaffolds were successfully modulated by changing the printing patterns. The average pore area approximatively ranged from 0.43–0.08 mm^2^ (Figure 2A). The 90_1 and 60_1 scaffolds were characterized by the highest pore area (i.e., 0.43 ± 0.04 mm^2^ and 0.42 ± 0.06 mm^2^, respectively, *p* > 0.05, and *p* < 0.05 vs. other scaffolds), as qualitatively proved by the previously shown morphology. The 45_1 and 90_0.7 type scaffolds had comparable pore sizes (i.e., 0.15 ± 0.12 mm^2^ and 0.16 ± 0.03 mm^2^, respectively; *p* > 0.05), which were higher than 60_0.7 and 45_0.7 type scaffolds (*p* < 0.05). The 60_0.7 and 45_0.7 type scaffolds showed the smallest pore areas (i.e., 0.08 ± 0.03 mm^2^ and 0.08 ± 0.06 mm^2^, respectively; *p* > 0.05). The high standard deviation in pore size of the scaffolds printed with 45° angle shift was given by the different shapes of pores obtained by printing with this angle shit, compared to the repeated motifs (i.e., squared, hexagonal, or triangular) observed in 90° and 60° angle shifts. Percent porosity values ranged from 55 to 67% (Figure 2B). Scaffolds printed with a 1.0 mm distance between filaments resulted to possess higher porosity than the scaffolds printed with a 0.7 mm distance (*p* < 0.05), while no differences were detected comparing the scaffolds printed with the same distance between filaments and different angle shifts (*p* > 0.05). The apparent density of the scaffolds also varied by changing the distance between the filaments, being the scaffolds printed with 0.7 mm distance were characterized by higher density values than scaffolds printed with 1.0 mm distance (Figure S4). 

Sterile scaffolds were then immersed in a culture medium and incubated at 37 °C, 5% CO_2_, to investigate the absorption of fluids inside the porous structure in simulated cell culture conditions. The percent weight variation of the scaffolds up to 3 weeks and the weight variation values at plateau are shown in Figure 2C. All the scaffolds showed a quick fluid absorption, namely, increasing weight variation values in the first 3 h after immersion in the culture medium, thus good hydrophilicity. At plateau, the scaffolds printed with 1.0 mm inter-filament distance demonstrated higher weight variation compared to that of the scaffolds printed with 0.7 mm inter-filament distance (Figure 2D; *p* < 0.05). 

**Figure 3:**
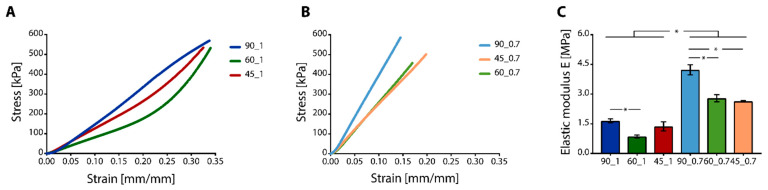
Compressive mechanical properties of 3D printed PU scaffolds. (**A**,**B**) Representative stress-strain curves of (**A**) samples printed with 1.0 mm distance, and (**B**) 0.7 mm distance between fibers, with 90°, 60°, and 45° angle shifts between subsequent printed layers. (**C**) Elastic modulus E was calculated from the compressive curves (* *p* < 0.05).

Representative stress-strain (*σ*-*ε*) curves obtained by compression tests for PU scaffolds printed with 1.0 and 0.7 mm distance are shown in Figure 3A,B, respectively.

Scaffolds printed with 0.7 mm inter-filament distance showed a linear response in the range of the applied stress, while scaffolds printed with 1.0 mm inter-filament distance showed a first linear region (i.e., *ε* < 0.2 mm∙mm^−^^1^) and subsequent non-linear response (i.e., *ε* > 0.2 mm∙mm^−^^1^). All the scaffolds proved to be able to support the applied loads with neither failure nor disruption up to 18 N. Elastic modulus (E) values of the scaffolds under compression ranged from 0.5–4.0 MPa, depending on the printed geometry and distance between filaments (Figure 3C). The scaffolds printed with 0.7 mm inter-filament distance resulted in higher E values, compared to those printed with 1.0 mm inter-filament distance between filaments (*p* < 0.05). This difference was given by the different number of intersection points between the printed filaments that sustained the load during compression. If the same surface area was considered, the scaffolds printed with filaments closer one to the other were characterized by a higher number of intersections between the filaments that were able to sustain the load, thus resulting in higher E values.

### 3.2. In Vitro Cytotoxicity of PU Scaffolds

**Figure 4:**
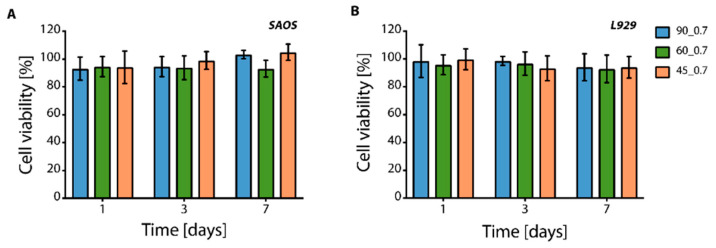
In vitro indirect cytotoxicity tests. Cell viability was intended as metabolic activity, measured for (**A**) SAOS-2 and (**B**) L929 cells cultured in a culture medium extracted by contact with PU samples with 0.7 inter-filament distance for 1, 3, and 7 days.

In vitro tests were conducted on scaffolds printed with a 0.7 mm inter-filament distance between filaments. Scaffolds printed with 1.0 mm inter-filament distance were excluded from the in vitro tests as they showed low E values (i.e., E < 1.5 MPa). In addition, high pore size due to the larger inter-filament distance hindered the pore colonization from seeded SAOS-2 and L929 cells (data not shown). In vitro indirect cytotoxicity showed that SAOS-2 cells (Figure 4A) and L929 cells (Figure 4B), used as osteosarcoma and connective tissue cell line models, respectively, displayed cell metabolic activity higher than 90%, thus proving the non-cytotoxicity of the printed PU scaffolds.

### 3.3. 3D In Vitro Bone Model

**Figure 5:**
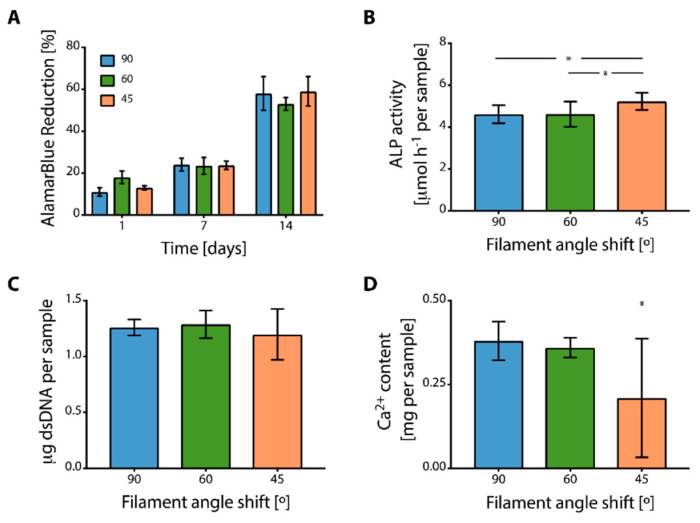
Characterization of the in vitro response of hMSCs osteo-differentiated on 90°, 60°, and 45° angle shift printed geometries with 0.7 inter-filament distance: (**A**) metabolic activity of cells over 2 weeks of culture, (**B**) content of DNA (cellularity) per sample (* *p* < 0.05), (**C**) alkaline phosphatase (ALP) activity, and (**D**) Ca^2+^ content (* *p* < 0.05).

After a preliminary cytotoxicity assessment was performed by using two different cell lines, the ability of the printed PU scaffolds in supporting primary bone cell adhesion and colonization to different scaffold inner geometries was tested by using hMSCs. All the scaffolds supported hMSC adhesion and an increase in metabolic activity was observed in time (Figure 5A). 

No statistical differences were observed comparing the cell viability (Figure 5A) and cellularity, namely the cell number in the scaffolds (Figure 5B), of hMSCs cultured on scaffolds with different geometries (*p* > 0.05). After 2 weeks of culture, ALP activity (Figure 5C) was significantly reduced for cells cultured on scaffolds with 90° and 60° angle shifts, compared to cells cultured on scaffolds with 45° (*p* < 0.05). Calcium content was higher for scaffolds printed with 90° geometry, compared to scaffolds printed with 45° geometry (Figure 5D).

**Figure 6:**
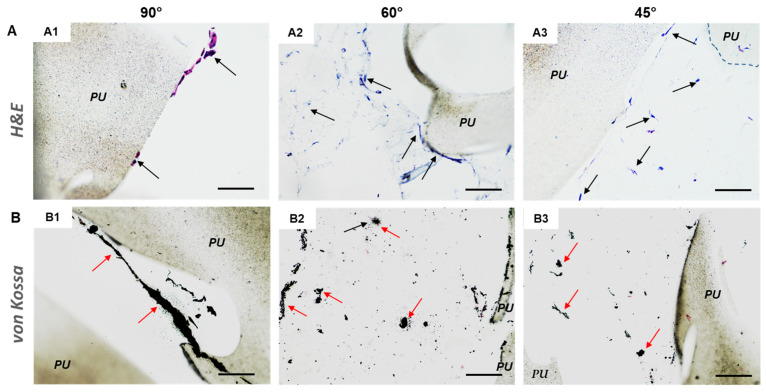
Histological staining on osteodifferentiated hMSC/scaffold constructs. 3D printed scaffolds consisted of PU fibers oriented at 90° (**A1**,**B1**), 60° (**A2**,**B2**), and 45° (**A3**,**B3**) angle shift printed geometries with 0.7 inter-filament distance. (**A**) H&E staining: cell nuclei are in blue-violet, cytoplasm in pink, black arrows = some representative cells; (**B**) von Kossa staining: mineralized matrix granules are in black, cell nuclei in red, red arrows = mineralized ECM. “PU” indicates polyurethane scaffolds; scale bar = 100 µm for all micrographs.

Histological analysis confirmed the presence of osteodifferentiated hMSCs colonizing the printed PU scaffolds. Figure 6 shows the H&E and von Kossa staining in the three scaffold types after being cultured with osteo-differentiated hMSCs, the latter revealing presence of mineralization nodules. It is important to outline that, unlike in 60° Figure 6(A2,B2) and 45° Figure 6(A3,B3), the cells cultured in 90° Figure 6(A1,B1) shift angle geometry scaffolds adhered only to the PU fibers and did not grow inside the pores.

**Figure 7:**
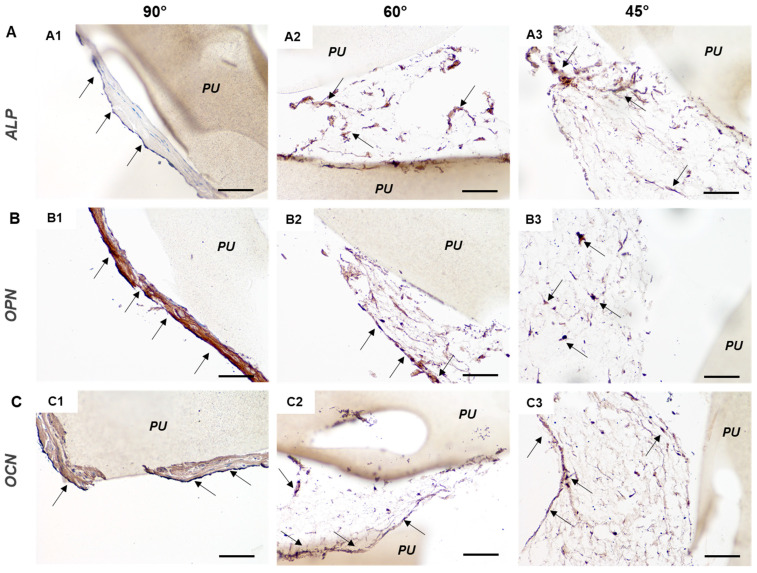
Immunohistochemistry of hMSCs osteo-differentiated on 3D PU scaffolds at 90° (**A1**,**B1**,**C1**), 60° (**A2**,**B2**,**C2**), and 45° (**A3**,**B3**,**C3**) angle shift printed geometries with 0.7 inter-filament distance. Representative micrographs revealing bone markers in brown and specifically the immunopositivity to (**A**) alkaline phosphatase (ALP), (**B**) osteopontin (OPN), (**C**) osteocalcin (OCN). “PU” indicates polyurethane scaffolds; arrows point to well visible cells; scale bar = 100 µm for all micrographs.

Bone markers were also revealed via immunohistochemistry to confirm hMSC osteo-differentiation (Figure 7). The early markers ALP Figure 7(A1–A3), osteopontin Figure 7(B1–B3), and osteocalcin Figure 7(C1–C3) were expressed on the printed PU scaffolds with different intensities. A good but not uniform positivity of intracellular ALP antigen was observed for 45° and 60° angle shift scaffolds, which was less but more uniformly observed for 90° angle shift scaffolds (Figure 7A). The expression levels confirmed the highest hMSC osteo-differentiation, in terms of intensity of late-stage marker expression, on 90° angle shift scaffolds Figure 7(A1,B1,C1).

**Figure 8:**
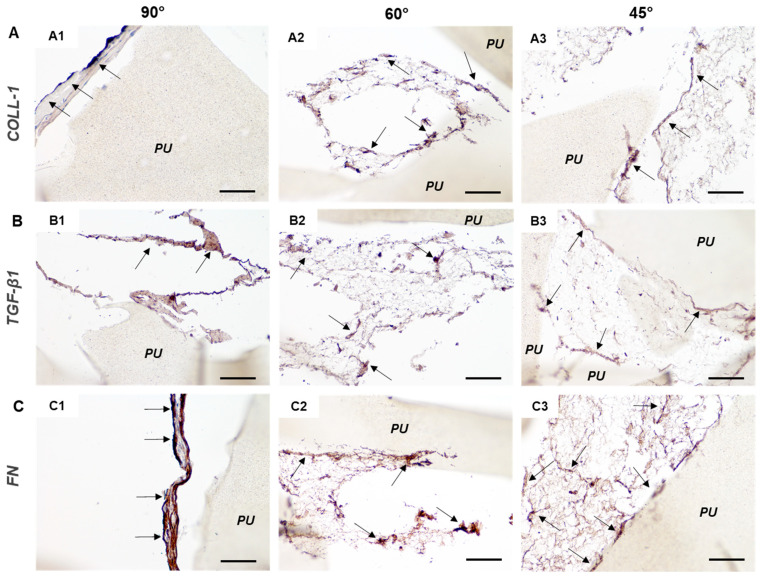
Immunohistochemistry of hMSCs osteo-differentiated on 3D PU scaffolds at 90° (**A1**,**B1**,**C1**), 60° (**A2**,**B2**,**C2**), and 45° (**A3**,**B3**,**C3**) angle shift printed geometries with 0.7 inter-filament distance. Representative micrographs revealing some ECM and signaling molecules in brown and specifically the immunopositivity to (**A**) collagen type I (COLL-1), (**B**) transforming growth factor beta 1 (TGF-β1), (**C**) fibronectin (FN). “PU” indicates polyurethane scaffolds; arrows point to well visible cells; scale bar = 100 µm for all micrographs.

Figure 8 shows the results of the immunohistochemical analysis in the hMSC/PU scaffold constructs for some key ECM molecules, such as collagen type I (Figure 8A) and fibronectin (Figure 8C), as well as a fundamental signaling molecule, TGF-β1 (Figure 8B), present in bone, which is considered to play a role in cancer progression and metastasis. Immunohistochemical analysis confirmed the absence of pore colonization in 90° angle shift PU scaffolds in Figure 7(A1,B1,C1) and Figure 8(A1,B1,C1). The cells adhered to the scaffold’s inner surfaces without being able to fill the scaffold pores. 

Collagen I, TGF-β1, and fibronectin were expressed in the three scaffold types. Collagen type I was revealed in all the three scaffold types, in particular in 60° and 45° angle shift PU scaffolds Figure 8(A2,A3). TGF-β1 was most intensely expressed in 90° angle shift PU scaffolds Figure 8(B1). Fibronectin was very intensely revealed in 90° angle shift PU scaffolds Figure 8(C1) and at the adhesion sites between cells and scaffold surfaces in 60° and 45° angle shift PU scaffolds Figure 8(C2,C3). All these findings supported the fact that the three PU scaffold types were cytocompatible and allowed for hMSC osteo-differentiation. The cells differentiated in 90° angle shift PU scaffolds showed higher expression of late bone markers than others, even though the scaffold colonization was not effective. The 60° angle shift PU scaffolds were thus used to generate the osteosarcoma model.

### 3.4. 3D In Vitro Osteosarcoma Model

**Figure 9:**
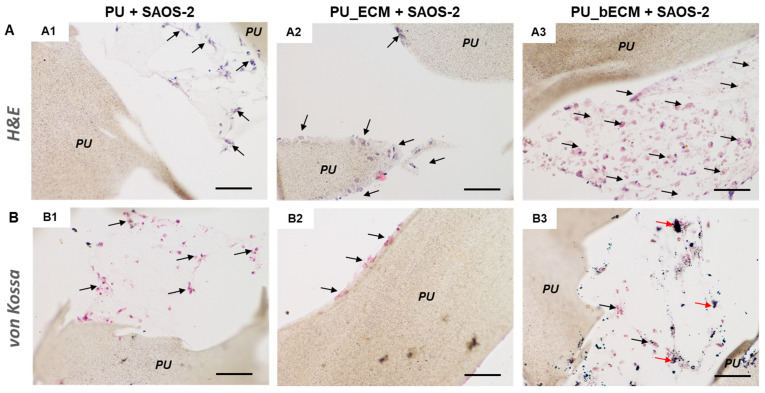
3D PU scaffolds with 60° shift angle geometry with 0.7 inter-filament distance as in vitro osteosarcoma model. Histological sections showing: (**A**) H&E staining; and (**B**) von Kossa staining of: (**A1**,**B1**) 3D printed PU scaffolds cultured with SAOS-2 cells; (**A2**,**B2**) 3D printed scaffolds containing immature (i.e., hMSC pre-generated) ECM cultured with SAOS-2 cells, and (**A3**,**B3**) 3D printed PU scaffolds containing mature (i.e., osteodifferentiated hMSC pre-generated) bone ECM cultured with SAOS-2 cells. Black arrows indicate SAOS-2 cells, red arrows indicate mineralized ECM granules, “PU” indicates polyurethane scaffolds; scale bar = 100 µm for all the micrographs.

The PU scaffolds printed with a 60° angle shift were then selected for the development of the in vitro osteosarcoma model (Table 2, see Discussion). The PU filaments arranged in these geometric features provided an optimal compromise between efficient pore colonization by hMSCs, and the highest calcium production. SAOS-2 cells were seeded on PU_bone ECM scaffolds, as well as on plain PU and PU_ECM scaffolds as controls (Figure 9). H&E staining revealed the presence of rounded cells, i.e., SAOS-2 cells, keeping contact with scaffold in all the analyzed samples (Figure 9A), particularly in the pores of scaffolds with pre-generated bone ECM Figure 9(A3). 

Von Kossa staining showed the presence of mineralized ECM only in the constructs constituted by scaffolds with pre-generated bone ECM and SAOS-2 cells Figure 9(B3). Different from the two controls in which SAOS-2 cells were layered on the polymeric surface Figure 9(B1,B2), SAOS-2 cells were detected in contact with the calcium nodules in the poral spaces Figure 9(B3). 

## 4. Discussion

3D in vitro osteosarcoma models are of paramount importance to deepen the knowledge on the development and progression of this cancer, as well as to ease and optimize the discovery of new treatments and drugs [3]. A reliable 3D in vitro model would allow the current limitations of 2D in vitro models to be overcome, as they oversimplify the physiological features of the pathological tissue [35]. On the other hand, such a 3D in vitro model would allow efficient management of the complex in vivo studies, without raising ethical concerns [6]. Developing reliable 3D in vitro cell/biomaterial constructs strictly depends on the fabrication of scaffolds with morphology (e.g., pore size and porosity) and mechanical properties suitable for the selected tissue, thus resulting in an adequately biomimetic microenvironment for cancer cell growth. Additive manufacturing technologies have demonstrated a unique potential to target these features [36]. In our study, the control of the morphological and mechanical features of the fabricated scaffold is achieved by using fused deposition modeling [37], while a chemically biomimetic microenvironment is artificially replicated in vitro by seeding hMSCs on the printed PU scaffolds, promoting their osteogenic differentiation and culturing osteosarcoma cells on the developed biomimetic 3D printed scaffold after hMSCs were removed by lysis. Under these circumstances, the osteosarcoma cells are cultured on a morphologically and mechanically suitable scaffold that contains the organic and mineral components of bone ECM produced by normal (i.e., non-cancerous) cells. 

PU is a class of polymers widely used in the biomedical field, both for clinical uses (e.g., cardiovascular applications [38]), which has been investigated to prepare scaffolds for bone regeneration [39], as well as in vitro bone models [16]. We selected PU as a polymer for the fabrication of our models due to its versatility, possibility of controlling its physicomechanical properties, non-cytotoxicity, and biostability, the latter being useful when long-term in vitro cultures are required since the same characteristics will be maintained on the long run. For these reasons, PU was considered a valuable polymer to prepare an in vitro osteosarcoma model. The commercial PU filaments were processed by fused deposition modeling, after optimizing the printing parameters, to obtain scaffolds with defined and reproducible architectures. Compared to other traditional fabrication technologies employed to prepare scaffolds for bone tissue engineering (e.g., particulate leaching [20], gas foaming [16], lyophilization [17]), the 3D printing technology used in this study allowed scaffolds with controlled pore size, distribution, and geometry to be obtained in a relatively short time [37,40]. Three patterns (i.e., 90°, 60°, and 45° angle shifts) and two different distances between adjacent printed filaments (i.e., 0.7 mm and 1.0 mm) were applied to fabricate scaffolds with different pore sizes and morphology, as these design parameters also served to modulate the physicomechanical properties of the scaffolds [41]. Tuning the design of the pores allowed the scaffold porosity to be modulated, which in turn influenced the mechanical properties and, eventually, the in vitro cell response. The distance between the printed filaments that we set was in the 700–1000 μm range, which led to pore size resembling the optimal pore dimension described in the literature for in vitro bone tumor models, namely, ranging from 200–300 μm [25], up to 1100 μm [42]. Different distances between filaments combined with diverse geometrical patterns resulted in distinct pore areas, that approximatively ranged from 0.40–0.08 mm^2^. Changes in the pore areas gave rise to a variation of the scaffold density, which, for all the prepared scaffolds, was in the trabecular bone density range. The porosity of the prepared scaffolds (55–67%) was comparable to the one achieved for scaffolds fabricated by additive manufacturing and used for bone regeneration [43], as well as for in vitro bone models [44].

The first step of this study addressed the identification of the most suitable scaffold type able to support normal bone tissue growth. We considered that osteosarcoma, as a primary bone tumor, develops within bone tissue. Preliminary in vitro tests using SAOS-2 and L929 cells showed that large pores of scaffolds printed with a 1.0 mm distance between filaments, resulted in poor cellularity and lack of pore occupation by cells and ECM molecules. In addition, those scaffolds had low rigidity. Thus, scaffolds printed with 0.7 mm inter-filament distance were further investigated as in vitro osteosarcoma models. Moreover, 1.0 mm inter-filament scaffolds demonstrated relatively low mechanical properties (i.e., E < 2 MPa), compared to 0.7 mm inter-filament distance scaffolds (i.e., E > 2 MPa). The mechanical properties of the scaffolds must be tuned to achieve a suitable biomimetic model, as they contribute to guiding cell fate and morphology [45,46], as well as the in vitro mimicry of the TME [47]. We tuned the mechanical properties of the scaffolds by designing their pore geometry and size [41]. Our scaffolds had Young’s modulus values in the order of MPa, which are superior [16,48], or comparable [49,50] to those described in literature able to mimic metastatic and primary bone tumors. Using the 0.7 inter-filament distance, we obtained bone-like constructs with similar cellularity using osteo-differentiated hMSCs. However, only in the 45° and 60° filament angle shift, the cells were able to colonize the poral spaces. In the 90° type, the cells resulted were laid onto the PU filaments, efficiently differentiated, but the pores were not populated. In the 45° type, we observed a less mature bone ECM generation, as the ALP activity was higher and calcium content lower. Therefore, the 60° filament angle shift was chosen as the optimal scaffold type. The selected material and fabrication technology used to design the morphology, physical, and mechanical properties, allowed us to define the most promising scaffold type (i.e., 60_0.7), which was subsequently used to prepare biomimetic in vitro models by incorporating different cell-secreted molecules.

The TME is made of the cellular and extracellular components in and by which the tumor exists and develops. TME is thus the result of dynamic and complex cell-cell and cell-ECM interactions taking place between normal and cancerous cells and their secreted molecules [4,13]. The TME consists of cancer cells, tumor stromal cells (e.g., stromal fibroblasts, endothelial cells, and immune cells), and ECM molecules (e.g., collagenic and non-collagenic molecules, hyaluronan, laminin). Among others, the role of collagen and its interaction with fibronectin is conditioned by tumor cells to enable signaling pathways and receptors that support cancer progression and metastasis. It is also considered that these phenomena are stimulated by the TME, as it promotes cancer cell heterogeneity and clonal evolution [51]. For instance, during tumor progression, cancer cells modify and divert the surrounding ECM towards the enhancement of anchorage-dependent molecules and the storage of pro-tumorigenic factors [52]. Polymeric scaffolds incorporating biomolecules, like gelatin, were able to stimulate fibroblast proliferation [53], as well as cancer cell interaction by metalloproteinase production [8], and integrin expression [54], which is associated with tumor progression [52]. Another signaling molecule involved in carcinogenesis is TGF-β, as in normal conditions, it possesses tumor-suppressing activity. Dysregulation of TGF-β has been associated with cancer invasiveness and metastasis [55]. Therefore, multidrug resistance often occurs because of the TME, which, by also recruiting normal cells to the scope, is revealed to be a protective environment for the tumor. To better target, the tumors, including osteosarcoma, drugs able to interact with the TME along with cell proliferation are a subject of study [56]. In this view, the mechanical consistency of scaffold-based in vitro models, particularly relevant for hard tissue tumors, maybe not be exhaustive. 

To better recapitulate the complexity of the TME, and specifically, the cancer cell-ECM cross-talk, we recreated some biological traits of the bone ECM within our 3D printed synthetic polymeric substrate. By cultivating hMSCs embedded in fibrin clots in the 60_0.7 scaffold type, followed by cell lysis, we generated the biomolecules proper of bone ECM, to be further explored as a microenvironment for osteosarcoma cell colonization. An immature (i.e., not mineralized, to be used as control) bone ECM was obtained by culturing undifferentiated hMSCs, whereas a mature (i.e., mineralized) bone ECM was obtained by differentiating the hMSCs towards the osteoblastic lineage for 3 weeks inside the PU scaffolds. This pre-generated bone ECM, as obtained in 3D printed PU 60_0.7, was analyzed via immunohistochemistry, resulting in rich collagen type I fibers, was positive for osteopontin, osteocalcin, fibronectin, TGF-β, and ALP, and via histochemistry, revealing calcium nodules. Quantitative tests corroborated the success of osteogenic differentiation by the presence of calcium [57]. The used procedure for cell lysis is known to preserve ECM molecules and growth factors [29]. In this way, we supported a suitable physicomechanical microenvironment, as given by the PU scaffolds, with a biological microenvironment, as given by cell-deposited bone-ECM. At this stage of the investigation, we aimed to have a simple model of SAOS-2 cells interacting with a bone ECM-like scaffold. As such, we did not co-culture normal (i.e., osteodifferentiated hMSCs) and cancerous (i.e., SAOS-2) cells together; however, this will be possible by removing the lysis step to save the generated osteoblasts [53]. Moreover, immune and/or vascular cells could be added [54,58]. The obtained findings revealed a remarkable interaction of SAOS-2 cells with the biohybrid scaffold. Indeed, SAOS-2 cells were able to colonize the pores of the scaffolds by interacting with the deposited bone ECM. This study aims to provide a model of improved complexity for osteosarcoma, enabling the study of drugs that target the TME. Combined drugs may disclose effective therapeutic options to improve the prognosis of osteosarcoma.

## 5. Conclusions

We 3D-printed PU scaffolds with reproducible patterns, geometries, and properties. The selected scaffolds (i.e., 0.7 mm distance between filaments, 60° angle shift) showed optimal porosity, density, cytocompatibility, and mechanical properties to be used for in vitro bone regeneration. The developed pre-culture condition (i.e., pre-seeding with osteo-differentiated hMSCs, subsequently lysed) was a suitable procedure to obtain a biohybrid scaffold, which combined the optimal physicomechanical properties of the 3D printed synthetic polymer, with the relevant biological properties of an in vitro pre-generated bone ECM. The developed bone-biomimetic 3D microenvironment was used for culturing SAOS-2 cells to create a TME recapitulating the main ECM features for the osteosarcoma study. The 3D in vitro model here developed could be used, in the future, to screen new therapeutics and to in-depth investigate osteosarcoma progression.

## Figures and Tables

**Table 1:** 3D printed polyurethane samples fabricated by varying the angle shift between subsequent layers (i.e., 90°, 60°, or 45°) and the distance between the center of filament printed in parallel on the same layer (i.e., 1.0 mm or 0.7 mm).

Filament Distance [mm]	Angle Shift [°]		
	90°	60°	45°
0.7	90_0.7	60_0.7	45_0.7
1.0	90_1	60_1	45_1

**Table 2:** 3D in vitro osteosarcoma models prepared using PU 60° pattern printed scaffolds.

3D Osteosarcoma Model Acronym	Scaffold Pre-Treatment (1st Step Culture, 3 Weeks)	Substrate Used for Osteosarcoma Cells	2nd Step Culture (1 Week)
**PU**	None	Plain scaffold	SAOS-2 cells
**PU_ECM**	Undifferentiated hMSC culture followed by cell lysis	Scaffold containing immature bone ECM	SAOS-2 cells
**PU_bECM**	Osteo-differentiated hMSC culture followed by cell lysis	Scaffold containing mature (mineralized) bone ECM	SAOS-2 cells

**Table 3:** Optimized 3D printing parameters used for the fabrication of 3D printed PU scaffolds as in vitro osteosarcoma models.

**Printing Parameters**	**Printing Speed** **[mm∙s^−1^]**	**Extrusion Temperature** **[°C]**		**Plate Temperature** **[°C]**		**Extrusion Multiplier**
	12	240		30		1.5
**Design parameters**	**Layer height [mm]**		**Filament thickness [mm]**		**Filament diameter [mm]**	
	0.4		0.4		1.7	

## Data Availability

Data are available upon request to the corresponding authors.

## Supplementary Materials

The following supporting information can be downloaded at: https://www.mdpi.com/article/10.3390/cancers14082003/s1, Figure S1: optimization of printing parameters; Figure S2: Representative SEM micrographs of printed PU scaffolds; Figure S3: representative microscopy images of the section of the scaffolds; Figure S4: apparent density of the scaffolds.
