# Supplementary material for: An Osteosarcoma Model by 3D Printed Polyurethane Scaffold and In Vitro Generated Bone Extracellular Matrix
Source: Cancers (Basel). 2022 Apr 15;14(8):2003. doi: 10.3390/cancers14082003 (PMC9025808; doi:10.3390/cancers14082003)
Supplement: Supplementary file 1 [file cancers-14-02003-s001.zip › cancers-1615552-supplementary.pdf]

cancers

MDPI

Supplementary Material

An Osteosarcoma Model by 3D Printed Polyurethane Scaffold and In Vitro Generated Bone Extracellular Matrix

Nicola Contessi Negrini [1,*], Claudio Ricci [2], Federica Bongiorni [1], Luisa Trombi [3], Delfo D'Alessandro [3], Serena Danti [2,*] and Silvia Farè [1]

[1] Department of Chemistry, Materials and Chemical Engineering "G. Natta", Politecnico di Milano, 20131 Milan, Italy; federica.bongiorni@mail.polimi.it (F.B.); silvia.fare@polimi.it (S.F.)
[2] Department of Civil and Industrial Engineering, University of Pisa, 56122 Pisa, Italy; claudio.ricci@med.unipi.it
[3] Department of Surgical, Medical, Molecular Pathology, University of Pisa, 56126 Pisa, Italy; l.trombi@yahoo.it (L.T.); delfo.dalessandro@unipi.it (D.D.)
* Correspondence: nicola.contessi@polimi.it (N.C.N.); serena.danti@unipi.it (S.D.)

Citation: Contessi Negrini, N.; Ricci, C.; Bongiorni, F.; Trombi, L.; D'Alessandro, D.; Danti, S.; Farè, S. An Osteosarcoma Model by 3D Printed Polyurethane Scaffold and In Vitro Generated Bone Extracellular Matrix. *Cancers* **2022**, *14*, 2003. https://doi.org/10.3390/cancers14082003

Academic Editors: Marilena Loizidou and Roberto Benelli

Received: 12 February 2022
Accepted: 11 April 2022
Published: 15 April 2022

Publisher's Note: MDPI stays neutral with regard to jurisdictional claims in published maps and institutional affiliations.

Copyright: © 2022 by the authors. Licensee MDPI, Basel, Switzerland. This article is an open access article distributed under the terms and conditions of the Creative Commons Attribution (CC BY) license (https://creativecommons.org/licenses/by/4.0/).

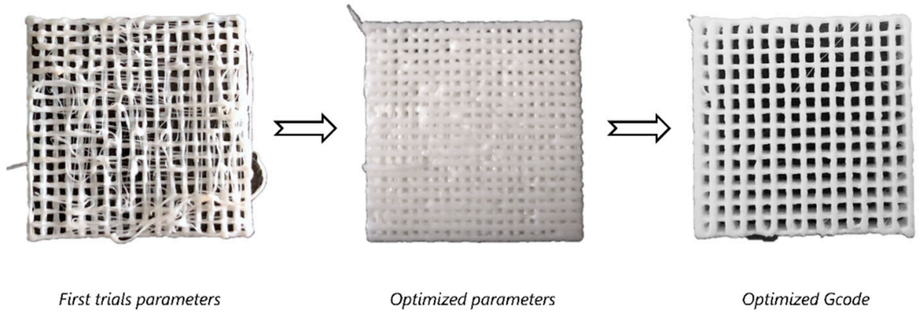

Figure S1. Optimization of the polyurethane printing process. Initial trials were performed to print samples, but clear defects can be macroscopically observed (left). Printing parameters were then optimized, but the movements of the printing head lead to undesired deposition of material (center). Finally, the G-code was optimized to obtain scaffolds with controlled printed geometry (right).

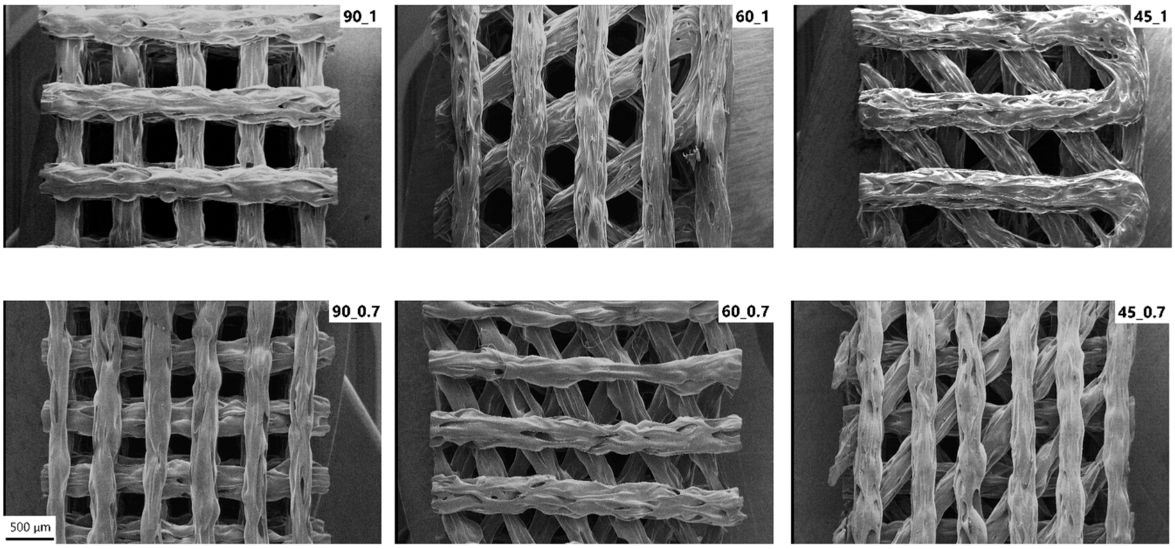

Figure S2. Representative scanning electron microscopy (SEM) micrographs of polyurethane scaffolds printed with different angle shifts between subsequent layers (i.e., 90°, 60° and 45°) and distance between filaments (i.e., 1.0 and 0.7 mm); scale bar = 500 µm. Samples were gold sputter-coated (Edward Sputter Coater S150B0) and imaged in secondary electron detection mode by StereoScan 360 (Cambridge Instruments).

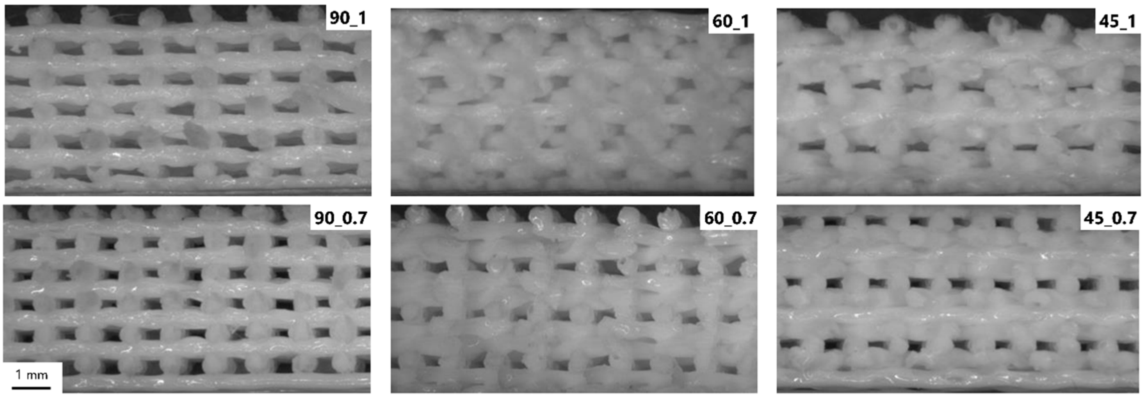

Figure S3. Representative images of the section of polyurethane scaffolds printed with different angle shifts between subsequent layers (i.e., 90°, 60° and 45°) and distance between filaments (i.e., 1.0 and 0.7 mm); scale bar = 1 mm.

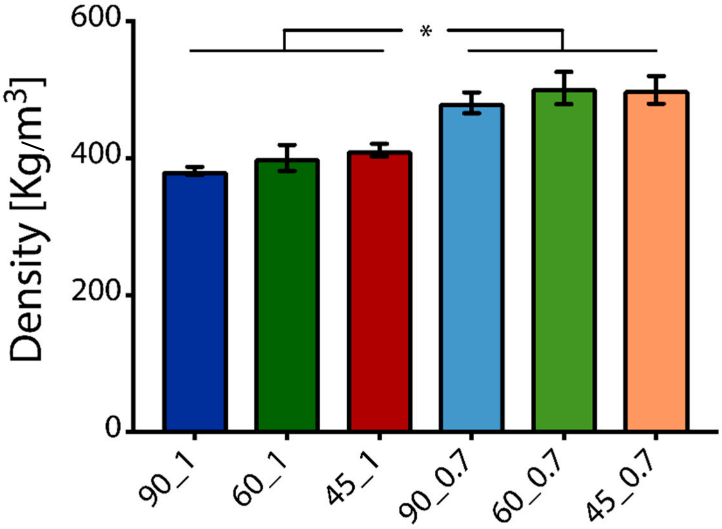

Figure S4. Average density of the printed polyurethane scaffolds measured as mass to volume ratio (* $p < 0.05$).
